# Par6 is an essential mediator of apoptotic response to transforming growth factor beta in NMuMG immortalized mammary cells

**DOI:** 10.1186/1475-2867-14-19

**Published:** 2014-03-01

**Authors:** Geordon Avery-Cooper, Meghan Doerr, Richard WD Gilbert, Mahmoud Youssef, Amy Richard, Patricia Huether, Alicia M Viloria-Petit

**Affiliations:** 1 Department of Biomedical Sciences, Ontario Veterinary College, University of Guelph, Room 3647, 50 Stone Road East, Guelph N1G 2 W1, ON, Canada

**Keywords:** TGFbeta, Par6, Apoptosis, Polarity, EMT, Integrins, Cell survival

## Abstract

**Background:**

We previously observed that the TGFbeta-Par6 pathway mediates loss of polarity and apoptosis in NMuMG cells. Here we investigate the contribution of Par6 versus TGFbeta receptor I activation to TGFbeta-induced apoptosis in association with changes in apico-basal polarity. We focus on the effect of Par6 activation on alpha6beta4 integrin expression and localization, and Nuclear Factor-kappaB (p65/RelA) activation, previously shown to mediate polarity-dependent cell survival.

**Methods:**

Using immunoblotting and/or immunofluorescence we investigated the effect of TGFbeta1 on apoptosis, alpha6, beta4 and beta1 integrin expression/localization, and p65/RelA phosphorylation/localization in monolayer and three-dimensional (3D) cultures of NMuMG cells with an overactive or inactive Par6 pathway. Results were quantified by band densitometry or as percent of 3D structures displaying a phenotype. Differences among means were compared by two-way ANOVA.

**Results:**

Blocking Par6 activation inhibits TGFbeta-induced apoptosis. Par6 overactivation enhances TGFbeta-induced apoptosis, notably after 6-day exposure to TGFbeta (*p* < 0.001), a time when parental NMuMG cells no longer respond to TGFbeta apoptotic stimuli. 48-hour TGFbeta treatment reduced beta4 integrin levels in NMuMG monolayers and significantly reduced the basal localization of alpha6 (*p* < 0.001) and beta4 (*p* < 0.001) integrin in NMuMG 3D structures, which was dependent on both Par6 and TGFbeta receptor I activation and paralleled apoptotic response. After 6-day exposure to TGFbeta, Par6-dependent changes to beta4 integrin were no longer apparent, but there was reduced phosphorylation of p65/RelA (*p* < 0.001) only in Par6 overexpressing cells. Differences in p65/RelA localization were not observed among the different cell lines after 48-hour TGFbeta exposure.

**Conclusions:**

Par6 and TGFbeta receptor I activation are both necessary for TGFbeta-induced apoptosis in NMuMG cells. Importantly, Par6 overexpression enhances the sensitivity of NMuMG to TGFbeta-induced apoptosis, notably upon prolonged exposure to this growth factor, when NMuMG parental cells are usually apoptosis-resistant. Thus, endogenous Par6 level might be important in determining whether TGFbeta will function as either a pro-apoptotic or pro-survival factor in breast cancer, and potentially aid in predicting patient’s prognosis and therapy response.

## Background

Transforming growth factor-beta (TGFβ) mediates mammary gland morphogenesis [1], and is known to play dual roles in breast cancer progression, by acting as a tumor suppressor in normal or pre-malignant cells, while promoting tumor progression in malignant cells [2,3]. TGFβ’s tumor suppressor function is in part mediated by its capacity to induce apoptosis, while its role in tumor progression has been linked to its ability to induce epithelial-to-mesenchymal transition (EMT), which promotes local invasion and metastasis [4,5].

TGFβ signals via a hetero-tetramer receptor formed by two TGFβ receptor I (TβRI) and two TGFβ receptor II (TβRII) units [6,7]. The canonical TGFβ/Activin signaling pathway is initiated by TGFβ binding to TβRII, which facilitates the formation of a complex with TβRI. Once in the complex, TβRII (a constitutively active kinase) phosphorylates and activates TβRI, which in turn recruits the receptor-activated Smads (R-Smads), Smad2 and Smad3. This leads to Smad 2/3 phosphorylation and activation by TβRI, enabling them to form a complex with the co-Smad, Smad4. The Smad2/3-Smad4 complex then translocates to the nucleus, where in cooperation with other transcription factors, co-activators, and co-repressors, modulates gene expression [8]. Apart from Smad signaling, TGFβ activates non-canonical pathways, including PI3K/Akt, NF-κB, Erk, p38 MAPK, JNK, FAK and the Par6 pathway, among others [9,10].

Partitioning-defective 6 (Par6) is part of the Par polarity complex that localizes to the tight junction (TJ) and is comprised of the three highly conserved proteins Par6, Par 3 and atypical protein kinase C (aPKC). In mammalian cells, this complex participates in the establishment of apico-basal polarity, directional migration, EMT (reviewed in [11]) and cell division [12]. Misregulation in expression and/or activity of Par complex components has been shown to promote breast cancer progression [13-16]. In epithelial mammary cells, Par6 is constitutively associated with TβRI at the TJ and is directly phosphorylated (at Ser345) and activated by TβRII in response to TGFβ. This is essential for TGFβ-induced EMT and facilitates metastasis [17,18]. The TGFβ-Par6 pathway promotes EMT via recruitment of the ubiquitin ligase Smurf1 to the TJ, leading to RhoA ubiquitination and its proteasomal degradation at the TJ site [17], the destabilization and dissolution of the TJ, and ultimately the rearrangement of the actin cytoskeleton [17,18]. Conversely, we also found Par6 to mediate TGFβ-induced apoptosis, one of TGFβ’s tumor suppressive effects [18]. The mechanisms of TGFβ-induced apoptosis are not fully understood, although transcriptional changes in pro- and anti-apoptotic proteins mediated by the Smad family [19], and Smad independent activation of TRAF6 and its downstream targets JNK and p38 MAPK [20,21] are well documented.

Here we further investigate the involvement of the TGFβ-Par6 pathway in apoptosis by focusing on its role in disrupting apico-basal polarity [17]. Growing evidence suggests that cell polarity modulates sensitivity to apoptosis. In particular, integrins, cell adhesion molecules that regulate cellular response to the extracellular matrix (ECM), were shown to promote cell polarity and confer resistance to apoptosis [22]. Specifically, the ligation of integrin α6β4 to reconstituted basement membrane was required for the polarization of mammary epithelial cells in three-dimensional (3D) culture [22], where cells display an apico-basal polarity similar to the mammary acinus *in vivo *[23]. This was shown to be dependent upon integrin ligation to laminin, which also conferred resistance to apoptosis-inducing stimuli via activation of NF-κB [22]. Autocrine laminin-5 ligation of α6β4 integrin was later shown to facilitate anchorage-independent survival of transformed mammary cells via activation of a Rac-NF-κB signaling cascade [24]. In agreement with the role of TGFβ in disrupting apical-basal polarity, TGFβ stimulation of mammary epithelial cells was shown to cause down-regulation of β4 integrin, and modulate the expression of many other integrins including α2, α5, β5, and α6 [25]. However, the impact that canonical and/or Par6 signaling has on apical-basal polarity and how it relates to integrin expression, integrin localization and apoptotic response to TGFβ has not been formerly addressed.

Here we used Namru murine mammary gland (NMuMG) epithelial cells displaying an overactive (Par6/wt) or inactive (Par6/S345A) Par6 pathway, or lacking β4 integrin, to investigate whether the TGFβ-Par6 pathway mediates changes in α6β4 integrin expression and/or localization, and whether these changes associate with loss of polarity and apoptotic response. We use NMuMG because we consider this to be --despite of its common description as “normal”-- the best characterized cell line that is representative of early stage (i.e., pre-malignancy) mammary transformation. Unlike other mammary cell lines available, TGFβ is able to induce both apoptosis and EMT in NMuMG cells [17,18,26], with apoptosis occurring only at earlier TGFβ exposure times (up to 5 days) in a susceptible fraction of the cells, while EMT predominates at later exposure times (over 5 days) [26] in the remaining, apoptosis-resistant population. This unique feature makes NMuMG cells an invaluable model to elucidate the specific signaling events that favor apoptosis versus cell survival/EMT in response to TGFβ. Important implications of addressing this question include the exciting possibility of potentiating cell death in advanced breast cancer subtypes, where TGFβ-induced EMT might play a role in metastatic spread and therapy resistance [27].

## Results

### Apoptosis of NMuMG treated with TGFβ1

We have previously shown that blocking Par6 activation suppresses loss of polarity and reduces apoptosis in response to TGFβ in 3D acini-like structures of NMuMG cells [18]. To confirm this, and to determine whether this phenomenon is restricted to cells growing as 3D structures, we evaluated apoptotic response to TGFβ1 in monolayers of NMuMG cells. For this purpose, we compared apoptotic response in NMuMG cells expressing the wild type form of Par6 (Par6/wt), which have been shown to display a constitutively active Par6 pathway [18], to NMuMG cells expressing a dominant negative form of Par6 (Par6/S345A), where Par6 activation is constitutively blocked [17,18].

**Figure 1:**
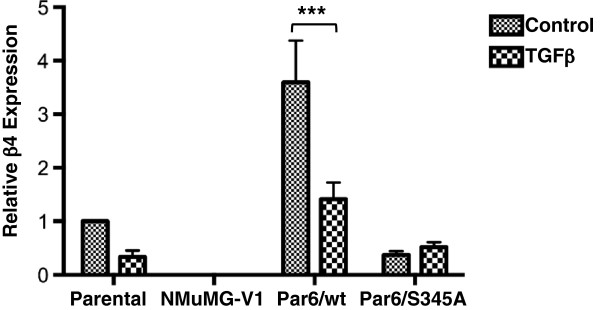
**β4 integrin expression in NMuMG cell variants.** Subconfluent NMuMG monolayers were treated with TGFβ1 (5 ng/ml) for 48 hours. Total RNA was extracted and reverse-transcribed to cDNA. β4 integrin was determined using quantitative real-time polymerase chain reaction (qRT-PCR) and normalized to GAPDH levels. Graph depicts the average fold change (± SEM) in Par6-expressing (Par6/wt and Par6/S345A) and an empty vector-expressing clonal variant of NMuMG cells (NMuMG-V1; later referred to as β4 null) relative to parental cells; n = 3 for biological replicates and n = 3 for technical replicates. Two-way ANOVA for all cell lines and treatments was significant (*p* < 0.0001 and *p* = 0.0060, respectively). Bonferroni post test compared differences between control and treatment for each cell line, ***p <0.0001.

Importantly, in preliminary experiments comparing the response of empty vector-expressing clonal lines to parental NMuMG cells (to ensure lack of bias due to the vector system used to express Par6) we came across an empty vector-expressing variant line (NMuMG-V1) that showed increased basal apoptosis, displayed a rapid EMT response to TGFβ and did not form polarized structures in 3D (Ozdamar and Viloria-Petit, unpublished observations). Since β4 integrin expression is required for the formation of polarized acini-like structures and to mediate cell survival in mammary epithelium [22] we examined the expression of β4 integrin mRNA in NMuMG-V1 as compared to Parental, Par6/wt and Par6/S345A cells with and without the addition of TGFβ, using qRT-PCR. We found the NMuMG-V1 cell line to be deficient in β4 integrin expression (Figure 1). It was also observed that the Par6/wt cells expressed significantly higher levels of β4 integrin as compared to parental cells and that TGFβ treatment downregulated β4 integrin mRNA expression in parental and Par6/wt cells but not in Par6/S345A (Figure 1). Based on these results we sought to compare the apoptotic response of all cell lines to TGFβ, and whether or not it correlated with the level of β4 integrin expressed by the cell lines. From here on we refer to NMuMG-V1 as “β4 null” cells, given their lack of β4 integrin expression.

Cell monolayers were treated with 5 ng/ml TGFβ1 for 48 and 144 hours (i.e., 2 and 6 days, respectively). The 48 hour time point was chosen based on our previous observation of this being a time at which apoptotic response can be detected in NMuMG cells [18]; while the 144 hours/6 days time point was chosen because NMuMG parental cells no longer undergo apoptosis at this time point [26]. To additionally investigate the relationship between the Par6 and the TβRI-activated canonical Smad pathway (i.e., the TGFβ/Activin pathway [28]) and their individual contribution to TGFβ-induced apoptosis, the TβRI inhibitor SB-431542 [29], known to block the TGFβ/Activin pathway and subsequent Smad2/3 activation [28] was included alone or in combination with TGFβ1. Relative caspase-3 cleavage (cleaved caspase-3 expression/caspase-3 expression) was determined to assess apoptosis.

**Figure 2:**
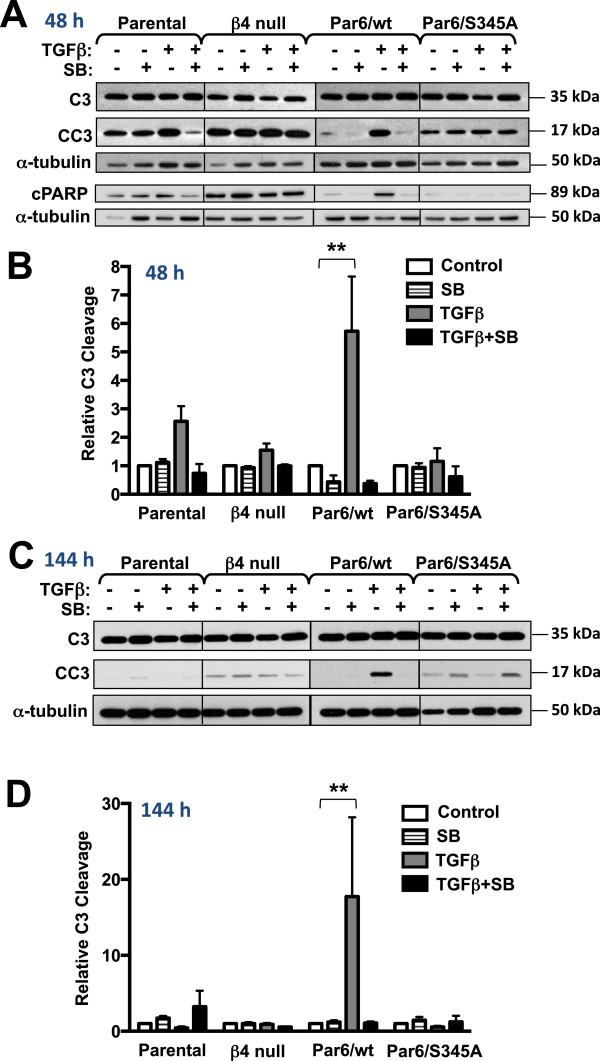
**Par6 overactivity sensitizes NMuMG cells to TGFβ-induced apoptosis.** Subconfluent monolayers of the indicated NMuMG–derived cell lines were treated with control media (DMSO alone), TGFβ1 (TGFβ; 5 ng/ml), the TβRI/SMAD inhibitor SB-431542 (SB) or TGFβ + SB. The level of cleaved caspase-3 was determined as a read-out of apoptosis after 48 hours **(A, B)** or 144 hours **(C, D)** treatment. **A** and **C** show levels of cleaved caspase-3 (CC3), uncleaved caspase-3 (C3) and α-tubulin (loading control) as determined by immunoblotting. Cleaved PARP (cPARP) in **A** served as an additional read-out of apoptosis. Graph in **B** and **D** represents the average relative caspase-3 cleavage calculated by band densitometry for 3 independent experiments. Relative C3 cleavage was calculated as the ratio of CC3 to C3. The value for DMSO control was set to 1 for each cell line. Two-way ANOVA for all cell lines and treatments was significant (*p* < 0.001). Bonferroni post test compared differences between control and treatment for each cell line, **p < 0.001.

Caspase 3 cleavage under basal conditions (in the absence of TGFβ1 and SB-431542) was higher in β4 null cells and lowest in Par6/wt cells at both time points tested (Figure 2A and C). Following 48 hours of TGFβ treatment, caspase-3 cleavage was increased in the parental NMuMG, β4 null, and Par6/wt cell lines as compared to basal levels, but not in Par6/S345A cells (Figure 2A,B). However, this effect was only significant (*p* < 0.001) in the Par6/wt cells (Figure 2B), suggesting that cells with an overactive Par6 pathway are more sensitive to TGFβ-induced apoptosis. There was an attenuated (two-fold lower) apoptotic response in the β4-null cell line compared to parental NMuMG cells, but it did not translate into a statistically significant difference between these two cell lines (Figure 2B). Examination of PARP cleavage as an additional indicator of apoptosis confirmed higher apoptotic response to TGFβ in Par6/wt cells at the 48-hour time point (Figure 2A).

Following TGFβ1 treatment for 144 hours, there was little to no detectable caspase-3 cleavage in the parental, β4 null, or Par6/S345A cells, while in the Par6/wt cells, there was a significant (*p* < 0.001) increase in caspase-3 cleavage (Figure 2C,D). SB-431542 inhibited the cleavage of caspase-3 (Figure 2A-D). These results indicate that both Par6 and TβRI activation are required for TGFβ-induced apoptosis. The lack of detectable increase in caspase-3 cleavage in the Par6/S345A expressing cell line suggests that Par6 activation, and not just Par6 expression, is required for TGFβ-induced apoptosis. Further, both basal and TGFβ-induced apoptosis after 48 hours treatment (Figure 2A,B) correlate with relative β4 integrin mRNA expression at the same time point (Figure 1).

### Effect of TGFβ on apoptosis in NMuMG three-dimensional structures

**Figure 3:**
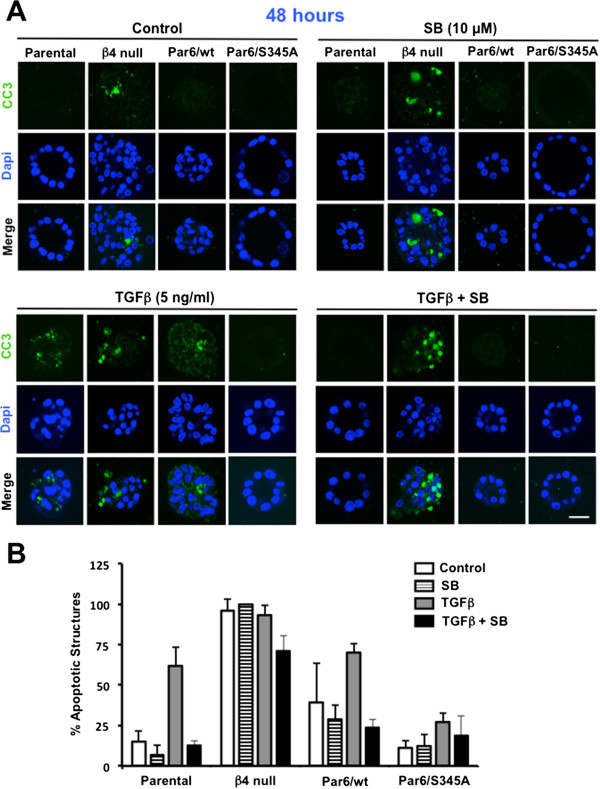
**TGFβ-induced caspase-3 cleavage requires both Par6 and TβRI activation in NMuMG 3D structures.** 12-day old 3D structures of the indicated NMuMG-derived cell lines were treated with control media (DMSO alone), TGFβ1 (TGFβ), the TβRI/Smad2 inhibitor SB-431542 (SB) or TGFβ + SB for 48 hours, fixed and immunostained for cleaved caspase-3 (CC3, green). Dapi was used to visualize the nuclei. **A**. Confocal images of representative structures for each cell line and treatment. Scale bar = 20 μm. **B**. Average percentage of apoptotic structures and error bars (SD) were calculated for n = 3 technical replicates within one experiment.

**Figure 4:**
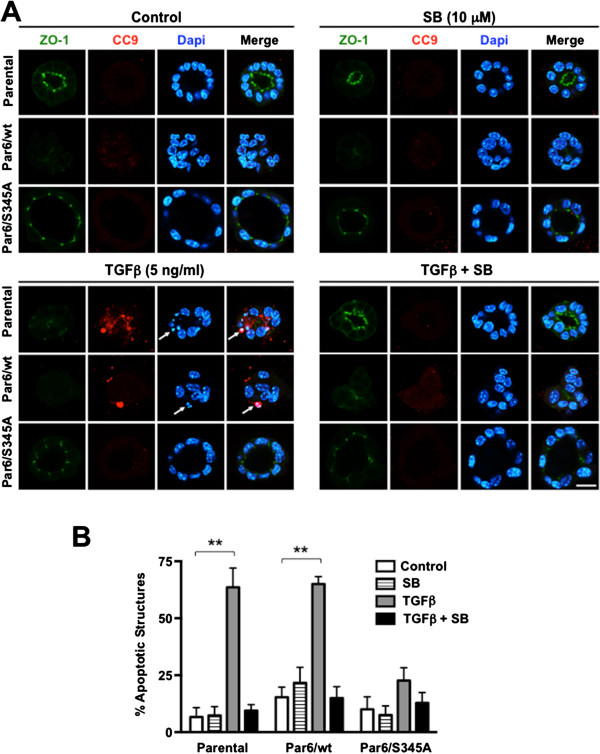
**TGFβ-induced caspase-9 cleavage requires both Par6 and TβRI activation in NMuMG 3D structures.** 12-day old 3D structures of the indicated NMuMG-derived cell lines were treated with control media (DMSO alone), TGFβ1 (TGFβ), the TβRI/Smad2 inhibitor SB-431542 (SB) or TGFβ + SB for 48 hours, fixed and immunostained for zonula occludens-1 (ZO-1, green) and cleaved caspase-9 (CC9, red). Dapi was used to visualize the nuclei. **A**. Confocal images show representative structures for each given cell line and treatment. Scale bar = 20 μm. Arrow = apoptotic nuclei. **B**. Average percentage of apoptotic structures was calculated per each cell type and treatment, with n = 3 for biological replicates. Two-way ANOVA for all cell lines and treatments was significant (*p* < 0.001). Bonferroni post test compared differences between control and treatment for each cell line, **p < 0.001.

**Figure 5:**
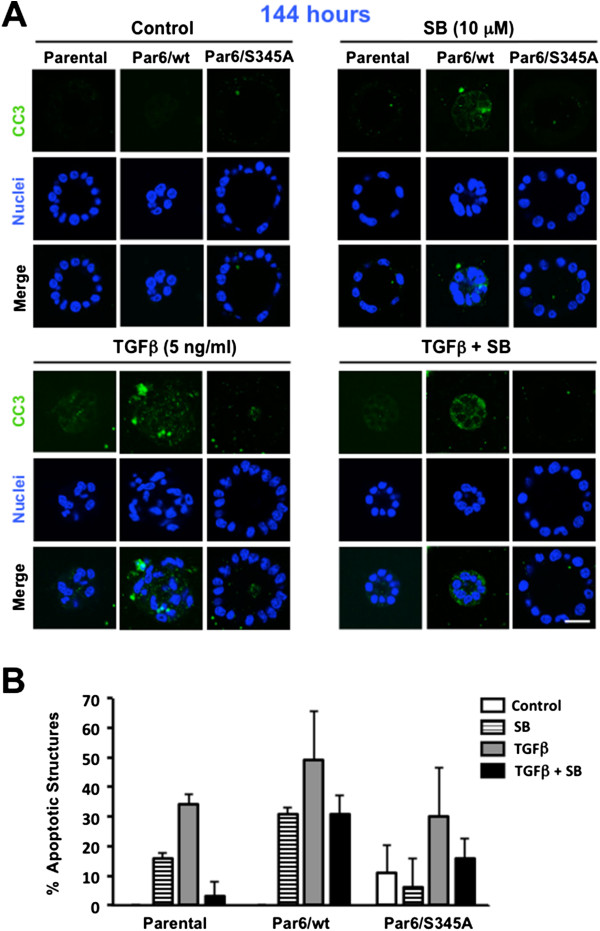
**Par6/wt-overexpressing cells maintain a high apoptosis response to TGFβ in long-term treated 3D culture.** 12–day old 3D structures of the indicated NMuMG-derived cell lines were treated with control media (DMSO alone), TGFβ1 (TGFβ), the TβRI/Smad2 inhibitor SB-431542 (SB) or TGFβ + SB for 144 hours, fixed and immunostained for cleaved caspase-3 (CC3, green). Dapi was used to visualize the nuclei. **A**. Confocal images show representative structures for each given cell line and treatment. Scale bar = 20 μm. **B**. Average percentage of apoptotic structures and error bars (SD) were calculated for n = 3 technical replicates within one experiment.

To confirm the effect of Par6 activation on TGFβ-induced apoptosis in conditions favoring the establishment of proper apico-basal polarity, we assessed the expression of cleaved/activated caspase-3 and cleaved/activated caspase-9 (an initiator of the intrinsic apoptotic pathway in response to TGFβ [30]), via immunofluorescence (IF) staining of NMuMG 3D structures grown on laminin-rich ECM (Figures 3, 4 and 5). The confocal images shown in Figures 3A, 4A and 5A show the most common phenotype observed for each cell line and treatment, while Figures 3B, 4B and 5B show plots that compare the average percentage of apoptotic structures for each cell line and treatment.

After treatment with DMSO alone (basal conditions) for 48 hours, Parental and Par6/S345A cells were generally acini-like, with obvious hollow-lumens and apical-lateral ZO-1, while β4 null and Par6/wt cells lacked lumens (Figures 3A, 4A). An average of 96% of the structures formed by β4 null cells were caspase-3 positive under basal conditions (Figure 3A,B), while for the other three cell lines only 12-39% of the structures were caspase-3 positive (Figure 3B). When caspase-3 and -9 activation were compared in these three cell lines, Par6/wt cells showed the highest basal percentage of caspase-3 (39%) and -9 (14%) positive cells (Figures 3B, 4B). Following TGFβ treatment, ≈ 60% of parental NMuMG structures lost polarity (evidenced by both, lack of a lumen and of apical-lateral ZO-1 staining) and showed immunoreactivity for both cleaved caspase-3 and -9. Par6/wt structures showed a similar apoptotic response to TGFβ (Figures 3 and 4). In contrast, the majority of Par6/S345A cells (≈ 75%) did not lose polarity in response to TGFβ treatment and showed no detectable levels of cleaved caspase-3 or -9 expression (Figures 3A, 3B, 4A and 4B). Statistical analysis for caspase-9 cleavage showed a significant increase (*p* < 0.001) in the number of parental and Par6/wt, but not Par6/S345A structures undergoing apoptosis in response to TGFβ treatment for 48 hours (Figure 4B). This effect was abrogated by SB-431542 treatment, indicating the requirement of both TβRI and Par6 activation for the loss of polarity and induction of apoptosis caused by TGFβ.

Caspase-3 cleavage immunostaining after a 144-hour TGFβ treatment, showed a 2-fold lower average of positive structures as compared to the 48-hour time point in parental cells (34% versus 62%; Figure 5B versus 3B), while the average of positive Par6/wt and Par6/S345A structures was similar at these two time points (66% versus 70% for Par6/wt and 30% versus 27% for Par6/345A at the 144- and 48-hour time points, respectively). Of note, the average percent of apoptotic structures at the 144-hour time point was at least 2-fold higher for Par6/wt as compared to the other two cell lines under all treatments, except for basal conditions. TβRI inhibition abrogated the induction of apoptosis in Parental cells, but was less effective at doing so in Par6/wt and Par6/S345A cells. β4 null cells were not analyzed at this time point because individual 3D structures were no longer identified. Taken together with our immunoblotting analysis, these results suggest that the Par6 pathway cooperates with the TGFβ/Activin signaling pathway to mediate apoptotic response to TGFβ, and Par6/wt overexpression promotes apoptosis upon prolonged exposure to TGFβ in NMuMG cells under both 2D and 3D culture conditions.

### Changes in integrin and E-cadherin expression in NMuMG following TGFβ treatment

**Figure 6:**
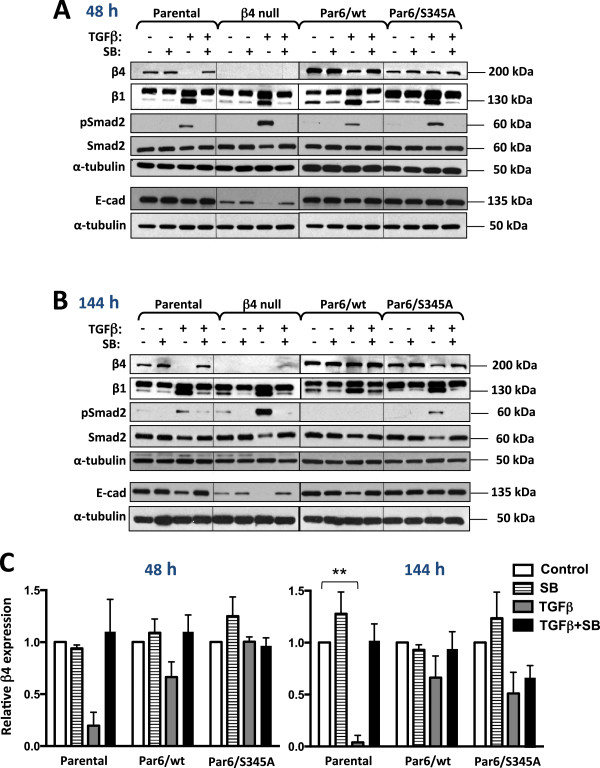
**TGFβ modulates integrin β4 expression in a Par6- and TβRI-dependent manner.** Subconfluent monolayers of the indicated NMuMG-derived cell lines were treated for 48 hours **(A)** or 144 hours **(B)** with control media (DMSO alone), TGFβ1 (TGFβ, 5 ng/ml), the TβRI/Smad2 inhibitor SB-431542 (SB, 10 μM) or TGFβ + SB. **A** and **B** show levels of β4 integrin (β4), E-cadherin (E-cad), β1 integrin (β1), phospho-Smad2 (pSmad2, Ser465/467) and Smad2 as determined by immunoblotting; α-tubulin was used as loading control. **C**. Graphs represent the average relative β4 integrin expression after 48-hour (left) or 144-hour (right) treatment as determined by band densitometry for n = 3 independent experiments. Relative β4 integrin expression was calculated as the ratio of β4 integrin to α-tubulin. The value for DMSO control was set to 1 for each cell line. Two-way ANOVA for all cell lines and treatments was significant (*p* < 0.05) for the 144-hour treatment. Bonferroni post test compared differences between control and treatment for each cell line, **p < 0.001.

To investigate whether changes in the expression of pro-survival integrins correlate with TGFβ-induced apoptosis and whether the Par6 or TGFβ/Activin pathway modulate these changes, we evaluated the expression of integrin α3, β1 and β4 following treatment for 48 or 144 hours with TGFβ1, SB-431542, or both in combination. The expression of α3 integrin was not significantly altered following TGFβ treatment at either of the two time points (data not shown). β1 integrin expression was induced by TGFβ at both 48 and 144 hours treatment (see 130 kDa band; Figure 6A,B). This induction was similar across all four NMuMG cell lines tested and was inhibited by SB-431542 treatment. Conversely, as previously observed at the mRNA level (Figure 1), TGFβ treatment down-regulated the expression of β4 integrin in NMuMG parental and Par6/wt cells following the 48-hour treatment, although neither difference was found to be statistically significant (Figure 6A and C, left graph). This down-regulation was inhibited by SB-431542 treatment and was not observed in Par6/S345A cells at this time point (Figure 6A and C, left graph). Following 144-hour TGFβ stimulation, β4 integrin expression was significantly decreased only in the parental cells (*p* < 0.001), while the decrease was non-significant in both Par6/wt and Par6/S345A cells (Figure 6B and C, right graph). Similarly to the 48-hour time point, SB-431542 treatment restored β4 integrin levels back to basal, particularly in parental and Par6/wt cells (Figure 6B and C, right graph).

To test whether changes in integrin expression correlated with changes in polarity proteins, we also examined E-cadherin expression, a marker of the adherens junctions (AJ). There was a slight decrease in E-cadherin following 48 hours TGFβ treatment in parental and Par6/wt cells (Figure 6A), which became more apparent at the 144 hours time point (Figure 6B). This effect was not seen in Par6/S345A (Figure 6A,B), in agreement with their reported inability to undergo loss of polarity and EMT in response to TGFβ [17,18]. β4 null cells expressed significantly lower basal levels of E-cadherin as compared to all other cell lines, and there was a pronounced decrease in E-cadherin expression in the β4 null cells following 48 hours and 144 hours of TGFβ treatment (Figure 6A,B). The decrease in E-cadherin expression observed in Parental, Par6/wt and β4 null cells following TGFβ treatment for 48 or 144 hours was abrogated upon inhibition of TβRI/Smad activation by SB-431542 treatment (compare E-cad and pSmad2 in Figure 6A and B). There was significantly higher TGFβ-induced Smad2 activation in β4 null cells as compared to all other cells. Taken together, these results suggest that β4 integrin downregulation depends on activation of TβRI, and to a lesser extend on Par6 activation, but only at the 48 hours time point. Both TβRI and Par6 activation are required for E-cadherin downregulation following TGFβ stimulation, in agreement with the demonstrated role of the TβRI/Activin and Par6 pathways in TGFβ-induced EMT [3]. The pronounced β4 downregulation observed in parental cells at the 144 hours time point, when these cells do not undergo significant apoptosis, suggests that a reduction in the expression level of β4 integrin is not likely to mediate apoptosis at this time point.

### Effect of TGFβ on α6β4 integrin localization in NMuMG three-dimensional structures

Enriched integrin expression at the cell’s basal site is a hallmark of apico-basal polarity and integrin α6β4 binding to laminin at the ECM was previously shown to signal survival in polarized, acini-like structures of mammary cells [22]. To investigate whether activation of the Par6 pathway could negatively impact survival signaling by promoting de-localization of integrins away from the basal site, we examined the expression of integrins α6β4 in 3D structures of Parental, Par6/wt and Par6/S345A NMuMG cells.

**Figure 7:**
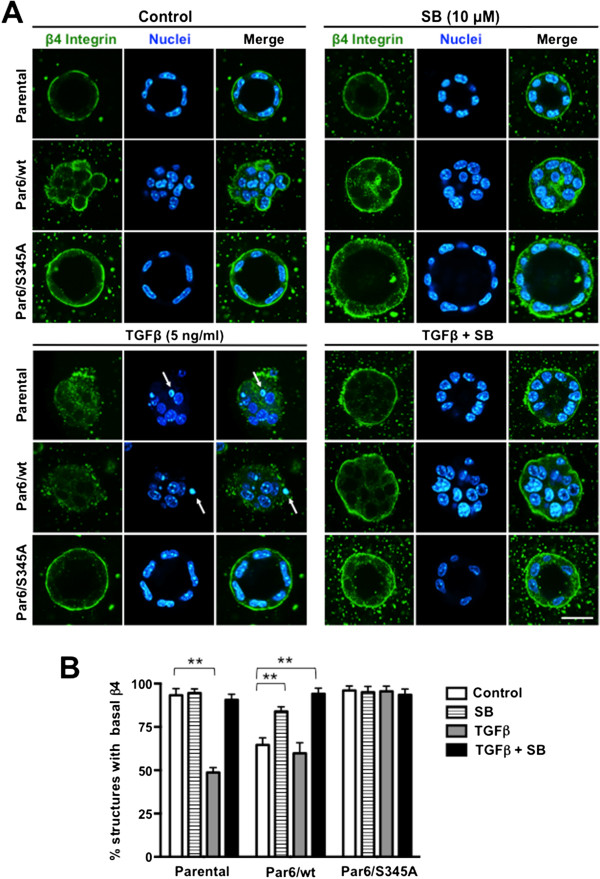
**Par6 and TβRI activation mediate TGFβ-induced loss of basal β4 integrin expression in NMuMG 3D structures.** 12-day old 3D cultures of the indicated NMuMG-derived cell lines were treated with control media (DMSO alone), TGFβ1 (TGFβ), the TβRI inhibitor SB-431542 (SB) or TGFβ + SB for 48 hours, fixed and immunostained for β4 integrin (green). Dapi was used to visualize the nuclei (blue). **A**. Confocal images show representative structures for each given cell line and treatment. Scale bar = 20 μm. Arrow = apoptotic nuclei. **B**. Average percentage of structures expressing basal β4 integrin were quantified for n = 3 biological replicates. Two-way ANOVA for all cell lines and treatments was significant (*p* < 0.001). Bonferroni post test compared differences between control and treatment for each cell line, **p < 0.001.

**Figure 8:**
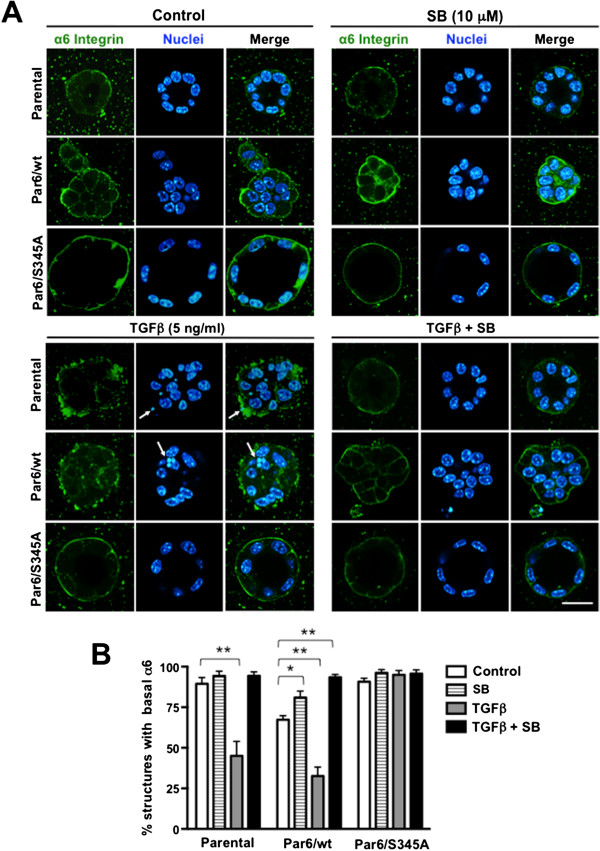
**Par6 and TGFβRI activation mediate TGFβ-induced loss of basal α6 integrin expression in NMuMG 3D structures.** 12-day old 3D cultures of the indicated NMuMG-derived cell lines were treated with control media (DMSO alone), TGFβ1 (TGFβ), the TβRI/Smad2 inhibitor SB-431542 (SB) or TGFβ + SB for 48 hours, fixed and immunostained for α6 integrin (green). Dapi was used to visualize the nuclei (blue). **A**. Confocal images show representative structures for each given cell line and treatment. Scale bar = 20 μm. Arrow = apoptotic nuclei. **B**. Average percentage of structures expressing basal α6 integrin were quantified for n = 3 biological replicates. Two-way ANOVA for all cell lines and treatments was significant (*p* < 0.01). Bonferroni post test compared differences between control and treatment for each cell line, *p < 0.05, **p < 0.001.

Both β4 and α6 integrin localize basally in mature, 14-day old parental NMuMG, Par6/wt, and Par6/S345A three-dimensional acini-like structures (Figures 7 and 8). 48-hour TGFβ treatment significantly decreased (*p* < 0.001) the number of parental structures expressing basal β4 integrin (Figure 7A,B), and the number of parental and Par6/wt structures expressing basal α6 integrin (Figure 8A,B). The decrease in basal expression of both α6 and β4 integrin observed in the parental structures, and of α6 integrin in the Par6/wt structures was abrogated by SB-431542 treatment. In contrast, the majority of Par6/S345A structures maintained basal expression of both β4 and α6 integrin after TGFβ treatment (Figures 7 and 8, respectively). Of note, SB-431542 treatment significantly increased the percent of Par6/wt cells expressing basal β4 and α6 integrin to levels similar to those observed in Parental and Par6/S345A 3D structures under basal conditions (Figures 7 and 8). All together, these results indicate that the change in integrin localization in NMuMG 3D structures is dependent on activation of both TβRI and the Par6 pathway.

### Assessment of the cell survival mediator NF-κB and its potential role in apoptosis downstream of the TGFβ-Par6 pathway

NF-κB signaling has been shown to promote cell survival downstream of α6β4 integrin ligation in polarized structures of mammary epithelial cells exposed to a variety of apoptotic stimuli [22]. Since NMuMG cells display proper distribution of a number of markers of apico-basal polarity in monolayer [17] as well as 3D cultures [18], we used monolayer cultures to investigate whether NF-κB mediates apoptotic resistance of Par6/S345A cells particularly after 48-hour treatment with TGFβ. At this time point, these cells do not downregulate β4 integrin expression and maintain basal localization of integrin α6β4, while the opposite is true for the apoptosis-sensitive Parental and Par6/wt cells.

**Figure 9:**
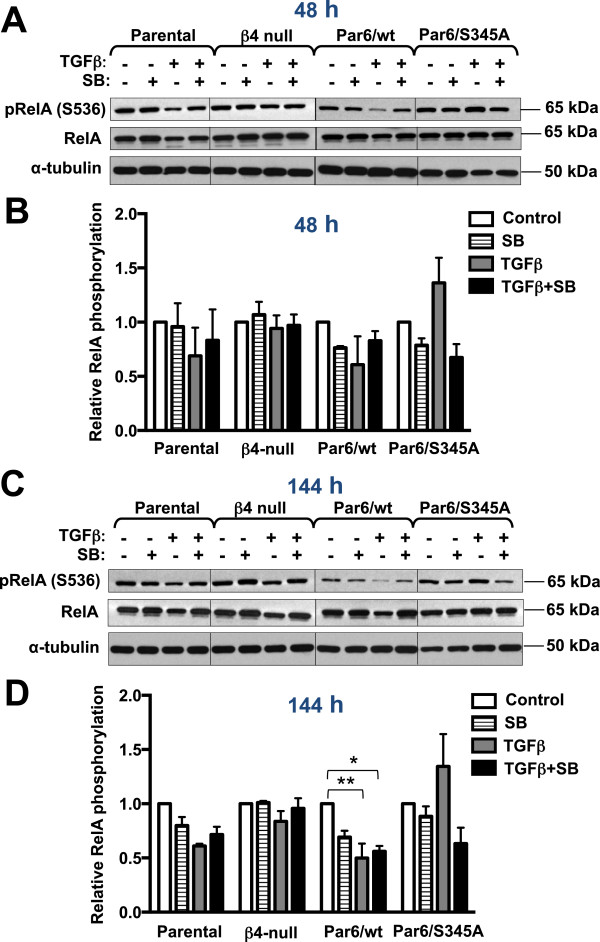
**TGFβ-induced changes in p65/RelA phosphorylation are dependent on Par6 and TβRI activation.** Subconfluent monolayers of the indicated NMuMG-derived cell lines were treated for 48 **(A, B)** or 144 **(C, D)** hours with control media (DMSO alone), TGFβ1 (TGFβ, 5 ng/ml), the TβRI inhibitor SB-431542 (SB, 10 μM) or TGFβ + SB. The level of phosphorylated p65/RelA (pRelA, S536), native p65/RelA and α-tubulin (loading control) was examined by immunoblotting. Graphs in **B** and **D** represent the average relative p65/RelA phosphorylation at S536 as determined by band densitometry for 3 independent experiments. Relative p65/RelA phosphorylation was calculated as the ratio of phosphorylated p65/RelA to native p65/RelA. The value for DMSO control was set to 1 for each cell line. Two-way ANOVA for all cell lines and treatments was significant (*p* < 0.01) only for the 144-hour treatment. Bonferroni post test compared differences between control and treatment for each cell line, *p < 0.05, **p < 0.001.

We first examined the phosphorylation status of p65/RelA (an essential component of the NF-κB complex) at Serine 536 (S536), which has been reported to be important for NF-κB transcriptional activity [31]. A decrease in p65/RelA phosphorylation, which paralleled a decrease in total p65/RelA level, was observed in parental and Par6/wt cells after both 48 and 144 hours of TGFβ exposure (Figure 9A, C). However, quantification of p65/RelA phosphorylation showed a significant TGFβ-induced decrease only in Par6/wt cells at the 144 hours time point (Figure 9B,D). In contrast, in response to TGFβ treatment, Par6/S345A cells showed a trend toward increased p65/RelA S536 phosphorylation, while phosphorylation at the same site remained relatively unchanged in β4 null cells at both time points (Figure 9B,D). In all TGFβ-treated cells, SB-431542 treatment restored phosphorylated p65/RelA to levels similar or slightly lower to those observed with SB-431542 treatment alone at both time points (Figure 9B,D). This suggest that changes in p65/RelA phosphorylation are dependent on both Par6 and TβRI activation, but in cells with an overactive Par6 pathway exposure to TGFβ over prolonged periods of time results in significant down-regulation of p65/RelA phosphorylation independent from TβRI activation (Figure 9D). Further, TβRI activation has a promoting rather than inhibiting effect on p65/RelA phosphorylation under the examined conditions.

**Figure 10:**
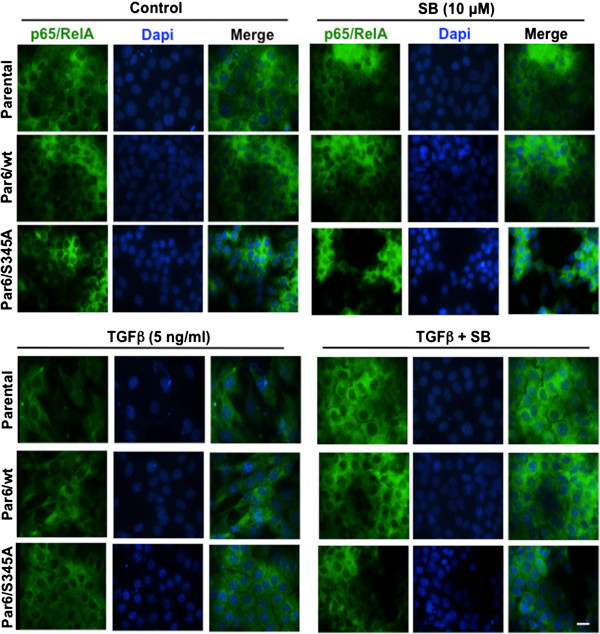
**NMuMG monolayers do not re-localize p65/RelA in response to 48-hour TGFβ treatment.** Subconfluent monolayers of the indicated NMuMG-derived cell lines were treated for 48 hours with control media (DMSO alone), TGFβ1, the TβRI inhibitor SB-431542 (SB) or TGFβ + SB. Cells were then fixed and p65/RelA expression/localization (green) was detected by immunofluorescence. Dapi was used to visualize nuclei (blue). Images are representative of two independent experiments, Scale bar = 20 μm.

Since p65/RelA transcriptional activity is known to depend on nuclear translocation [32], next we examined its localization after 48-hour TGFβ stimulation using IF imaging. We chose to analyze 48-hour treated cell monolayers, because TGFβ-induced changes in the level of native and phosphorylated p65/RelA, which paralleled apoptotic response in both parental and Par6/wt cells, were seen in whole lysates obtained from monolayer cell cultures at the 48-hour time point. p65/RelA localization was always cytoplasmic and no change in this localization was observed in any of the aforementioned cell lines upon TGFβ exposure (Figure 10), suggesting that a reduction in NF-κB transcriptional activity does not mediate TGFβ pro-apoptotic effect on NMuMG cell monolayers, at least at this time point.

## Discussion

The TGFβ-Par6 pathway was initially reported to be required for the loss of the TJ and TGFβ-induced EMT in NMuMG cells [17]. We have previously demonstrated the role of this pathway in invasiveness and metastasis of breast cancer cells [18], when we also observed that blockade of Par6 activation abrogates TGFβ-induced loss of polarity of acini-like structures of NMuMG and inhibits TGFβ-induced apoptosis [18]. By comparing caspase-3 and -9 activation in NMuMG cells with active or inactive Par6 and TGFβ/Activin signaling pathways, this study validates our previous findings [18] and provides new insights on the interplay between Par6 and TβRI activation in determining mammary cell apoptotic response to long or short-term TGFβ exposure.

The similar capacity of the dominant negative Par6/S345A mutant to block TGFβ-induced apoptosis in 2D (monolayer) as well as 3D cultures on rBM indicates that this phenomenon is not restricted to acini-like structures, and the effect of both Par6 and TβRI inhibition in blocking TGFβ-induced apoptosis supports the notion that activation of both TβRI and Par6 is required for apoptosis to occur. These findings are in agreement with the demonstrated role of TβRI in recruiting and activating Smad2/3 and TRAF6 to signal apoptosis [19-21,33]. With regard to Par6, they suggest a potential link between the stability of cell-cell junctions, cell polarity and apoptosis, which has also been supported by previous studies. For instance, treatment of prostate cancer cells with aurothiomalate was shown to disrupt the PKCι/Par6 complex, leading to caspase-3 activation and apoptosis [34]. Similarly, radiation-induced injury was shown to promote apoptosis via disruption of the Cdc42/Par6/atypical protein kinase C Par polarity complex that localizes to the TJ [35], and Par3 knockout and consequent withdrawal from the Par complex promoted apoptosis in keratinocytes [36]. Taken together, these and our observations suggest that perturbations of the Par complex and the TJ leads to apoptosis. The possible disruptive effect of Par6 phosphorylation on Par6’s interaction with other members of the Par complex has yet to be determined and could potentially explain the positive impact of Par6 activation on TGFβ-induced apoptosis.

The connection between apico-basal polarity and cell survival has also been reported. Weaver et al. demonstrated that polarized acini-like structures of mammary epithelial cells are resistant to a variety of apoptosis-inducing stimuli [22], supporting the notion that loss of polarity might be a pre-requisite for cells to undergo apoptosis. This and further studies by the Weaver’s group reiterated the importance of α6β4 integrin and NF-κB signaling in driving survival of both normal and transformed mammary epithelial cells [22,24]. This evidence prompted us to investigate the potential connection between activation of the Par6 pathway, α6β4 integrin expression/localization and NF-κB signaling in the context of TGFβ-induced apoptosis. Apart from our previous findings pointing to the requirement of Par6 signal for apoptotic response to TGFβ [18], we were intrigued by the high apoptosis rate shown by an empty vector-expressing NMuMG cell variant previously generated by the Wrana group [17], which failed to form acini-like structures on rBM and had very high levels of basal apoptosis (Ozdamar and Viloria-Petit et al., unpublished observations and Figure 3). Here we show that these cells lack expression of β4 integrin, express significantly lower basal levels of E-cadherin and display increased Smad activation in response to TGFβ, a group of features that correlate with their inability to form polarized acini-like structures and with their high apoptosis rate in both monolayer and 3D culture. Further, despite of their high basal apoptosis and high Smad activation in response to TGFβ, these cells have reduced apoptotic response to this growth factor. Taken together, these results indicate a potential link between β4 integrin-mediated apico-basal polarity, TGFβ signaling and apoptosis.

We found that TGFβ1 stimulation for 48 hours reduces expression of β4 integrin, and disrupts basal localization of α6β4 integrin in 3D structures of NMuMG cells. Because these effects were not seen in cells with an inactive Par6 pathway or Parental cells treated with a TβRI inhibitor, both of which maintained ZO-1 and E-cadherin expression, these results suggest that the modulation of α6β4 integrin by TGFβ requires both activation of Par6 and of TβRI, and that the activity of these two signaling effectors is also essential for loss of polarity. Our results are also in agreement with a previous report showing that TGFβ downregulates β4 integrin expression in mammary epithelial cells [37].

Although we were not able to detect changes in p65/RelA localization in response to TGFβ stimulation for 48 hours, we observed a reduction in p65/RelA expression and concomitant downregulation of p65/RelA phosphorylation that was rescued by TβRI inhibition in both Parental and Par6/wt cells. This effect was more pronounced at the 144-hour time point, when it became statistically significant and independent of TβRI activation only for Par6/wt cells. Because TGFβ was not able to downregulate p65/RelA phosphorylation in β4 null cells our results suggest that TGFβ’s impact on p65/RelA phosphorylation may require β4 integrin expression. Based on the contrasting increase in phospho-p65/RelA observed in Par6/S345A in response to TGFβ, and the capacity of the TβRI inhibitor to block this increase as well, we speculate that TβRI activation, which is more prominent when the S345 phosphorylation site on Par6 is blocked [18] (see also pSmad2 level as a read-out of TβRI activity in Figure 6A and B), promotes p65/RelA phosphorylation. Consequently, it is probable that the donwregulation of phospho-p65/RelA seen in Par6/wt cells at the 6-day time point is the result of prolonged preferential activation of Par6 over TβRI. Therefore, the balance between Par6 and TβRI activation might be key in modulating the activation status of signaling pathways downstream of the TGFβ receptors and hence the cellular effects of TGFβ.

Given that prolonged (144-hour) exposure to TGFβ results in significant changes in p65/RelA phosphorylation in Par6/wt cells, the only cells that undergo significant apoptosis at this time point, it is still possible that negative modulation of NF-κB signaling in Par6/wt cells plays a role in the higher apoptotic response of these cells to long-term TGFβ exposure. Thus, more extensive studies looking at NF-κB localization, NF-κB-dependent promoter activity and expression of target genes upon short- and long-term exposure to TGFβ are necessary to ultimately establish or rule-out the involvement of this transcription factor in TGFβ-induced apoptosis, particularly in the context of Par6 activation.

## Conclusions

In summary, here we show that Par6 and TβRI activation are both necessary for TGFβ-induced apoptosis in NMuMG cells. Par6 overactivation significantly enhances NMuMG cells sensitivity to TGFβ-induced apoptosis, notably upon prolonged exposure to this growth factor in monolayer culture, when NMuMG parental cells are usually insensitive to TGFβ’s pro-apoptotic effect. Given that TβRI activation in Par6/wt expressing cells under these conditions appears significantly reduced (judging by the level of Smad activation), this suggests that a high ratio of Par6 to TβRI activation upon long-term TGFβ exposure can revert NMuMG from apoptosis resistant to apoptosis sensitive. Both Par6 and TβRI signaling are required for loss of apical basal polarity and for the reduction in β4 integrin expression, loss of basal localization of integrin α6β4, and downregulation of NF-κB p65/RelA expression in response to 48-hour stimulation with TGFβ. Of note, long-term TGFβ exposure (144 hours) results in significant reduction in p65/RelA phosphorylation via Par6 activation in contrast to increased p65/RelA phosphorylation via TβRI activation. Establishing the contribution of NF-κB and other mediators of cell survival signaling to TGFβ’s capacity to induce apoptosis might prove useful in stratifying breast cancer patients for conventional or molecular-targeted therapy. In this regard, it will be important to determine whether in those advanced breast cancers that display active TGFβ signaling, higher endogenous Par6 levels correlate with better patient prognosis due to enhanced TGFβ-dependent tumor suppression and/or improved therapy response.

## Methods

### Antibodies, growth factors, and inhibitors

Antibodies included: β1 integrin (#610167), β4 integrin (#553745), α6 integrin (#555734) (all from BD Biosciences); Smad2 (#3103), phospho-Smad2 (S465/467, #3101), NF-κB p65 (#4764), phospho-NF-κB p65 (S536, #3033), E-cadherin (#4065), β-actin (#4967), Caspase-3 (#9662), Cleaved Caspase-3 (#9661), Cleaved Caspase-9 (#9509), cleaved PARP (#9544) (all from Cell Signaling), α-tubulin (#T5168, Sigma-Aldrich), ZO-1 (#sc-33725, Santa Cruz), and Alexa-Fluor®-conjugated secondary antibodies (Molecular Probes®, Life Technologies). Growth factors/hormones included: rhTGFβ1 (Invitrogen) and insulin (Sigma-Aldrich). The TβRI inhibitor SB-431542 was from InvivoGen.

### Cell lines and culture conditions

NMuMG parental cells (ATCC) were grown in high glucose DMEM (HyClone) supplemented with 10% FBS and 10 μg/ml insulin. NMuMG cells expressing Pmep5 (Vector), Pmep5-mPar6 (Par6/wt), or Pmep5-mPar6 mutant S345A (Par6/S345A) (a generous gift from Dr. J. Wrana, SLRI, Toronto, ON) were previously generated [17] and grown in DMEM high glucose supplemented with 10% FBS, 10 μg/ml insulin, and 500 μg/ml G418. All cells were maintained in a humidified incubator at 37°C in the presence of 5% CO_2_ and 95% atmospheric air.

### Matrigel 3D cultures and immunofluorescence staining

NMuMG cells were maintained under standard culture conditions as aforementioned. Subconfluent monolayers were trypsinized in a solution of 0.05% Trypsin/0.53 mM EDTA (Invitrogen), washed once with DMEM plus 10% FBS, resuspended in assay media, and plated as a single cell suspensions on 100% growth factor reduced Matrigel (BD Biosciences) using the overlay method as previously described [18]. Assay media contained 2% Matrigel added to mammary epithelial growth media supplemented with 0.4% bovine pituitary extract, 10 ng/ml epidermal growth factor (EGF), 5 μg/ml insulin and 0.5 μg/ml hydrocortisone, according to manufacturer’s instructions (PromoCell). Medium was changed every 3 days. 5 ng/ml recombinant human TGFβ1 and/or 10 μM of the TGFβ receptor I inhibitor SB-431542 was added after mature structures were formed (12-15 days after cell plating) and replenished every 2 days. Immunofluorescence (IF) was performed as previously described [18]. Briefly, 3D cultures on 4-well glass chamber slides (BD Biosciences) were washed twice with ice-cold PBS, after which cultures were fixed with 4% Paraformaldehyde (EMS) in PBS for 20 minutes at room temperature. The fixed cultures were then washed with PBS and permeabilized with cold 0.5% Triton-X (Bio-Rad) in PBS for 10 minutes, followed by four washes with 100 mM Glycine (Fisher) in PBS. Permeabilized cultures were blocked for one hour at room temperature using 10% goat serum diluted in IF buffer, which consisted of 0.1% BSA (Santa Cruz), 0.2% Triton-X (Bio-Rad), and 0.05% Tween-20 (Bio-Rad) dissolved in PBS. Fixed cultures were next incubated with primary antibody diluted in blocking solution overnight (final dilutions as recommended by manufacturer). Next day, 3D structures were washed three times in IF buffer and then incubated with Alexa Fluor® secondary antibodies (Life Technologies; final dilution 10 μg/ml). Following incubation with secondary antibody, cultures were washed three times in IF buffer, followed by a ten-minute incubation with 0.3 μM 4′, 6′-diamidino-2-phenylindole (DAPI) (Sigma-Aldrich) in PBS. The chambers were then removed, and slides were mounted with coverslips using Prolong Gold® (Promega) to preserve the fluorescence. All slides were analyzed using an Olympus FV500 confocal microscope (Olympus). Images were captured using Fluoview 5.0 software (Olympus).

### Treatment and western blotting

For analysis of protein expression, confluent (to be used for 2 day treatment) or sub-confluent (to be used for 6 day treatment) cells were serum starved with reduced serum medium (high glucose DMEM plus 2% FBS) for 2 hours, which was then changed to the reduced serum medium containing TGFβ1 (5 ng/ml) and/or the SB-431542 inhibitor (10 μM). Medium was changed every 2 days. Cells were lysed with protein lysis buffer (20 mM Tris-HCl, 150 mM NaCl, 1 mM Na_2_EDTA, 1 mM EGTA, 1% Triton, 2.5 mM sodium pyrophosphate, 1 mM β-glycerophosphate, 1 mM Na_3_VO_4_, 1 μg/ml leupeptin) (Cell Signaling) supplemented with 1 mM PMSF (Sigma-Aldrich), 2 μg/ml aprotinin (Sigma-Aldrich), 1 mM Na_3_VO_4_ (New England Biolabs), and 1% phosphatase inhibitor cocktail 2 (Sigma-Aldrich). Protein samples were resolved on 7.5%, 10%, or 12% polyacrylamide (Bio-Rad) gels and then transferred to a PVDF membrane (Roche), washed twice with Tris Buffered Saline plus 0.05% Tween (TBS-T) and then blocked for one hour with either 5% milk in TBS-T or 5% BSA (to detect phosphorylated proteins) in TBS-T. Membranes were incubated with primary antibody diluted in blocking solution overnight (final dilution as recommended by manufacturer, except for pSmad2, used 1:500; Smad2, used 1:5,000; and αTubulin, used 1:160,000). Next day, membranes were washed 3 times per 10 min with TBS-T, and then incubated with either 1:10,000 goat anti-mouse HRP, 1:10,000 mouse anti-rabbit HRP (Both Sigma-Aldrich), or 1:1,000 goat anti-rat HRP (Millipore) diluted in 5% milk in TBS-T for 1 hour at room temperature. Membranes were then washed 3-6 times per 10 min in TBS-T following secondary antibody incubation. Immunoglobulin-antigen complexes were incubated with a chemiluminescence detection system (Roche) for 1 min and subsequently exposed to X-ray film (Kodak) or to a Chemidoc™ MP Image System (Bio-Rad). Protein bands from film were quantified using a FluorChem 9900 imaging system (Alpha Immunotech), while images captured with Chemidoc MP were quantified using Image Lab software (Bio-Rad). Protein loading was normalized using bands for α-tubulin. Relative phosphorylation levels were normalized to native protein bands.

### Quantitative real-time PCR (qRT-PCR)

RNA was isolated from cultured cells by homogenization in Trizol at cold temperature followed by the Aurum total isolation RNA kit (Bio-Rad). cDNA synthesis was performed using the iScript cDNA synthesis kit (Bio-Rad) with 1 μg total RNA. Appropriate quality control steps were included along the way conforming to the MIQE guidelines [38,39]. The cDNA that was obtained was diluted 1:4 in nuclease free water and 4 μl of diluted cDNA was added to reaction mixes (10 μl) containing 5 μl Sso Fast Eva Green Supermix (Bio-Rad) and 500 nM of each primer. Primer sequences were obtained from the Harvard primer bank: mouse GAPDH F 5-cacaccgaccttcaccatttt-3; mouse GAPDH R 5-gagacagccgcatcttcttgt-3; mouse β4 F 5-aggcctgagaacagaggtca-3; mouse β4 R 5-ccggagatgcacattgtatg-3. Primers were optimized using a temperature gradient and eight point standard curve to determine PCR efficiency. Acceptable efficiency was deemed between 90% and 110%. qRT-PCR amplifications were carried out using a CFX-96 (Bio-Rad) as follows: an initial denaturation step 2 minutes at 95°C, followed by 40 cycles of 5 seconds at 95°C and 5 seconds at 59°C. Data was expressed as relative gene expression normalized to GAPDH mRNA, which was determined to be a suitable housekeeping gene using qBase Plus software (BioGazelle).

### Evaluation of cell death and integrin localization in 3D cultures

Apoptotic cells were detected by IF staining with cleaved caspase-3 or cleaved caspase-9 (Cell Signaling) antibodies. Integrin localization was assessed by IF staining for α6 and β4 integrin. Results were quantified as percent of apoptotic structures or percentage of structures expressing basal α6 or β4 integrin. 3-10 low power (20×) confocal images, or the equivalent to at least 100 structures, were analyzed per group and condition for each experiment.

### Statistical analysis

Means were calculated and plotted along with standard error bars. All statistical analyses were done using GraphPad Prism software version 3.1 (GraphPad). Data were first analyzed by two-way ANOVA. Significant differences between means were subsequently determined using the Bonferroni post-tests and were considered statistically significant when the *p* value was less than 0.05. Three biological replicates were included for all experiments, with the exception of cleaved PARP western blotting and cleaved caspase-3 IF, which were tested in two and one independent experiments, respectively. qRT-PCR experiments additionally included three technical replicates per run.

## Abbreviations

AJ: Adherens junction; BSA: Bovine serum albumin; C3: Caspase-3; CC3: Cleaved caspase-3; CC9: Cleaved caspase-9; Cdc42: Cell division control protein 42 homolog; DAPI: 4*'*, 6*'*-Diamidino-2-phenylindole; DMEM: Dulbecco’s modified eagles medium; E-cadherin: epithelial cadherin; ECM: Extracellular matrix; EGF: Epidermal growth factor; EMT: Epithelial-to-mesenchymal transition; ErbB2: Human epidermal growth factor receptor 2/HER2; Erk: Extracellular signal-regulated kinase; FAK: Focal adhesion kinase; FBS: Fetal bovine serum; HRP: Horseradish peroxidase; IF: Immunofluoresence; JNK: c-Jun N-terminal kinase; lrBM: Laminin-rich basement membrane; NF-κB: Nuclear factor-Kappa B; NMuMG: Namru murine mammary gland; p38 MAPK: p38 mitogen-activated protein kinase; Par3: Partitioning-defective 3; Par6: Partitioning-defective 6; Par6/S345A: Par6C mutant (Serine 345 mutated to Alanine); Par6/wt: Par6C wild type; PBS: Phosphate-buffered saline; PI3K/Akt: Phosphoinositide-3 Kinase/v-Akt (viral AKR mouse thymoma) homolog; PMSF: Phenylmethanesulfonyl Fluoride; PVDF: Polyvinylidine fluoride; qRT-PCR: Quantitative real-time polymerase chain reaction; rBM: Reconstituted basement membrane; SD: Standard deviation; TGFβ: Transforming growth factor-beta; TβRI: Transforming growth factor-beta receptor type I; TβRII: Transforming growth factor-beta receptor type II; TJ: Tight junction; ZO: Zonula occludens.

## Competing interests

The authors declare that they have no competing interests.

## Authors’ contributions

GA-C performed the majority of experiments presented in this manuscript (Figures 2, 4, 6, 7, 8 and 9) and prepared the initial draft of the manuscript. MD performed the cleaved PARP immunoblotting, and cleaved caspase-3 IF and imaging (Figures 2, 3 and 5). RWDG performed the real time PCR analysis shown in Figure 1 and participated in manuscript preparation. MY and AR performed the NF-κB immunofluorescence presented in Figure 10. PH performed the western blots for Caspase 3 presented in Figure 2. AMV-P supervised the research and prepared the final version of the manuscript. She also participated in experiments involving culture, treatment, IF, and imaging of NMuMG 3D structures. All authors read and approved the final manuscript.
